# N-acetyl-cysteine attenuates remifentanil-induced postoperative hyperalgesia via inhibiting matrix metalloproteinase-9 in dorsal root ganglia

**DOI:** 10.18632/oncotarget.15217

**Published:** 2017-02-09

**Authors:** Yue Liu, Yuan Ni, Wei Zhang, Yu-E Sun, Zhengliang Ma, Xiaoping Gu

**Affiliations:** ^1^ Department of Anesthesiology, Affiliated Drum Tower Hospital of Medical School of Nanjing University, Nanjing 210008, Jiangsu Province, China

**Keywords:** remifentanil-induced hyperalgesia (RIH), matrix metalloproteinase-9 (MMP-9), N-acetyl-cysteine (NAC), dorsal root ganglia (DRG)

## Abstract

Treatment of remifentanil-induced postoperative hyperalgesia (RIH) remains a clinical challenge because the mechanisms are not fully understood. Matrix metalloproteinase-9 (MMP-9) is a key component in neuroinflammation because of its facilitation of pro-inflammatory cytokine maturation. Therefore, inhibition of MMP-9 may represent a novel therapeutic approach to the treatment of RIH. Sprague-Dawley rats were randomly divided into three groups: Control, Incision and Remifentanil. A right plantar surgical incision was performed in Group Incision, and intraoperative remifentanil (0.04 mg/kg, 0.4 ml) was infused subcutaneously for 30 min in Group Remifentanil. The results indicated that intraoperative remifentanil induced an up-regulation and activation of MMP-9 in DRGs but not spinal cords. MMP-9 was expressed primarily in DRG neurons co-expressing mu opioid receptors (MOR), and elicited interleukin-1β (IL-1β) cleavage in DRG neurons and satellite glial cells (SGCs). Intraperitoneal injection of N-acetyl-cysteine (NAC), a broadly used safe drug, significantly attenuated RIH via suppressing the activation of MMP-9 in DRGs. NAC inhibited the cleavage of IL-1β in DRGs, which is a critical substrate of MMP-9, and markedly suppressed glial activation and neuron excitability in spinal dorsal horn induced by remifentanil. These results demonstrated that NAC can effectively alleviate RIH via powerfully inhibiting MMP-9 activation in DRGs.

## INTRODUCTION

Remifentanil is commonly administered intravenously during general anesthesia. However, it has contrary pro-nociceptive actions and promotes postoperative hyperalgesia [1, 2]. The underlying mechanisms remain unclear, a safer and more effective treatment remains to be found.

The mechanisms of remifentanil-induced hyperalgesia (RIH) might be related to spinal N-methyl-d-aspartate receptor (NMDAR)-dependent central sensitization [3, 4]. Previous studies indicated that remifentanil-induced postoperative mechanical allodynia and thermal hyperalgesia was associated with evaluated phosphorylation of NMDAR subunit 2B (NR2B) in spinal dorsal horn [4, 5]. During the process of RIH, the increase in pro-inflammatory cytokines, including interleukin-1β (IL-1β), IL-6, and tumor necrosis factor α (TNF-α), released from activated astrocyte and microglia, directly activated neuronal NMDAR and facilitated neuronal plasticity [6, 7]. NMDAR antagonists, such as ketamine, MK-801 and Ro 25-6981, prevented the development of RIH in a rat model of incisional pain [4, 8, 9]. Furthermore, administration of selective chemokine (C-X-C motif) receptor 2 (CXCR2) antagonist SB225002, glycogen synthase kinase-3β (GSK-3β) inhibitor TDZD-8, cyclin-dependent kinase 5 (Cdk5) inhibitor roscovitine, or alpha-7 nicotinic acetylcholine receptor (α7-nAchR) agonists PHA-543613 can attenuate RIH via regulating the expression and function of spinal NMDAR [5, 10–12]. Unfortunately, a safe drug, which is effective on RIH and can be used in the clinic is not yet available.

Matrix metalloproteinases (MMPs) are a group of calcium-dependent zinc-containing endopeptidases that are responsible for the cleavage of most extracellular matrix (ECM) proteins and cytokines [13]. The excessive proteolytic activity of MMP-9 and MMP-2 in central and peripheral nervous system, mediates ECM abnormality, and leads to a variety of neuropathologic conditions, including neuroinflammatory response [14, 15], disruption of the blood-brain barrier [16], and peripheral as well as central hypersensitivity that participate in hyperalgesia [17]. Specifically, MMP-9 and MMP-2, produced in injured dorsal root ganglia (DRG) neurons, may enhance neuronal transmission and produce neuropathic pain via triggering microglia activation and phosphorylating NR1 and NR2B in neurons in spinal cord [18]. Acute morphine induced a significant up-regulation of MMP-9 in primary sensory neurons expressing MOR in DRGs, masking morphine-induced analgesia through MMP-9-dependent activation of satellite glial cells (SGCs) and increase in IL-1β [19, 20]. The evidence mentioned above suggested that MMP-9 and MMP-2 may be involved in opioids-mediated nociception. However, it is unclear whether MMP-9 and MMP-2 also contribute to RIH.

In the present study, we examined the activity of MMP-9 and MMP-2 in spinal cord and in DRGs after subcutaneous infusion of remifentanil in a rat model of incisional pain, and further investigated the underlying mechanisms and sought a safe treatment for RIH using N-acetyl-cysteine (NAC). NAC, an ‘old’, safe and commonly used clinically medicine [21], is a donor of cysteine and prevents the cysteine residue on MMP-9 and MMP-2 from being oxidized, which is vital to MMPs activation [22, 23]. Considering its safety and potential ability to interfere with the vital process of the ‘cysteine switch’ during MMPs activation, we chose NAC to target MMP-9 and MMP-2.

## RESULTS

### Intraoperative remifentanil infusion induced postoperative hyperalgesia and increased MMP-9 activity in DRGs

**Figure 1:**
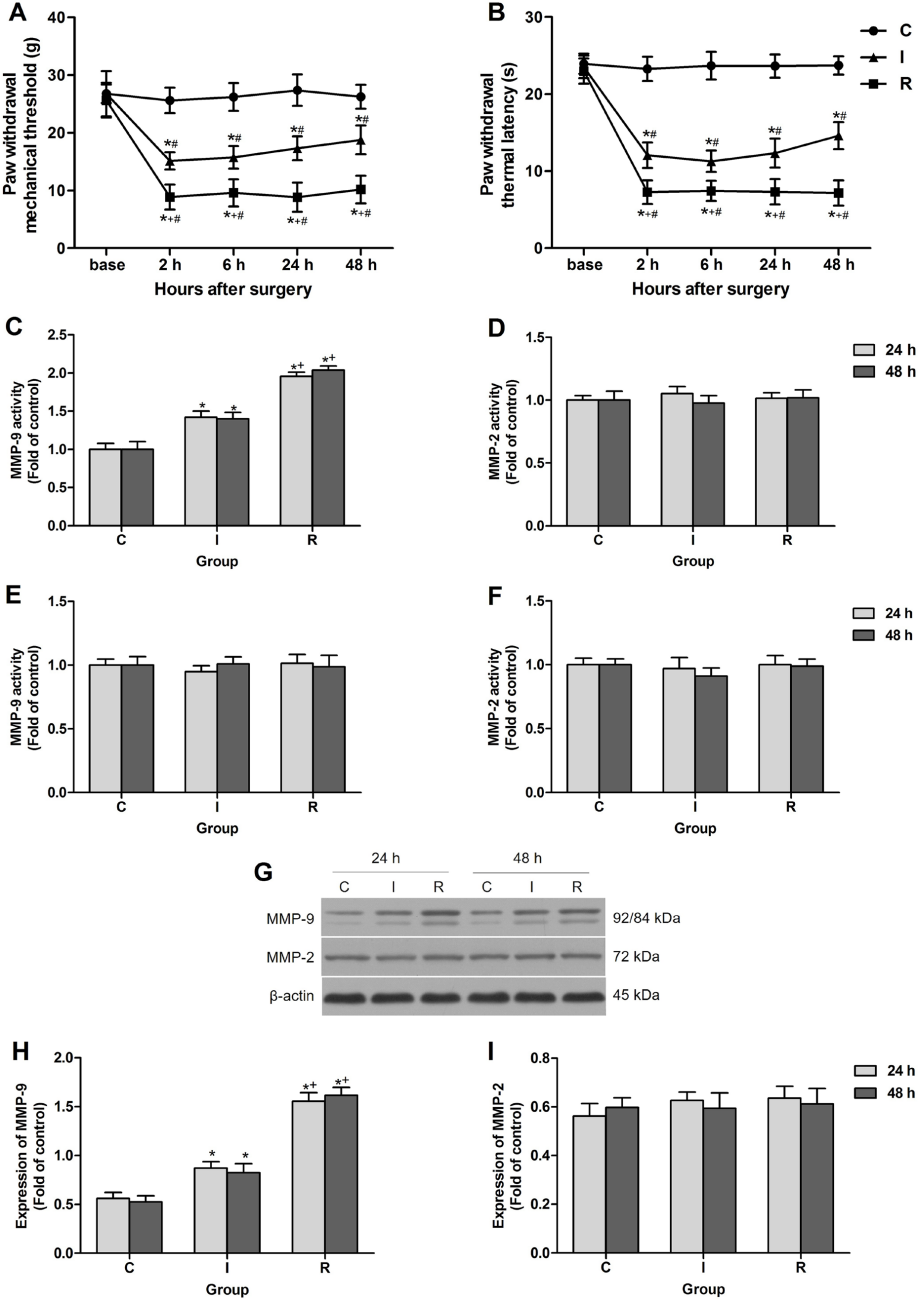
Intraoperative subcutaneous remifentanil infusion increased MMP-9 activity and expression in ipsilateral DRGs (**A** and **B**) Remifentanil-induced postoperative mechanical allodynia presented as PWMT and PWTL of right hind paw (*n* = 8). (**C** and **D**) Colorimetric quantitative detection showed that MMP-9 was significantly activated in ipsilateral lumbar DRGs at 24 h and 48 h after intraoperative remifentanil infusion, and the activity of MMP-2 remained unchanged (*n* = 5). (**E** and **F**) Neither MMP-9 nor MMP-2 activity was changed in ipsilateral spinal cord dorsal horn at 24 h and 48 h after surgery (*n* = 5). (**G–I**) Western blotting showed that the expression of MMP-9 was up-regulated in ipsilateral lumbar DRGs at 24 h and 48 h after intraoperative remifentanil infusion, and MMP-2 remained unchanged. Representative bands for MMP-9, MMP-2 and β-actin resulted in products of 92/84, 72, 43 kDa (G) and data summary (H and I) are shown (*n* = 5). β-actin was used as a loading control. Values expressed as mean ± SD. Group C: sham surgery; Group I: subcutaneous infusion of saline during incisional surgery; Group R: subcutaneous infusion of remifentanil during incisional surgery. Significant difference in pain behaviors was revealed after Repeated measures ANOVA, and significant difference in the results of western blotting and Colorimetric quantitative detection was revealed after One-way ANOVA (*P < 0.05 compared with group C, ^+^*P* < 0.05 compared with group I, ^#^*P* < 0.05 compared with baseline, Bonferroni post hoc tests).

There was no significant difference in the baseline of PWMT and PWTL among groups before surgery. Compared with the baseline, the administration of sevoflurane and the subcutaneous infusion of saline for a period of 30 min to rats in the absence of plantar incision in group C did not produce significant changes in PWTL or PWMT. However, compared with baseline and group C, the plantar incision evoked a decrease in PWMT and PWTL in the operated hind paw after surgery at all time points observed (*P* < 0.0001). Intraoperative infusion of remifentanil significantly enhanced mechanical allodynia and thermal hyperalgesia induced by the plantar incision. This was manifested by a significant decrease in PWMT (*P* < 0.0001) and PWTL (*P* < 0.0001 at 2 h, 24 h and 48 h, *P* = 0.00014 at 6 h) in group R compared with rats in group I (Figure 1A, 1B).

The activity of MMP-9 and MMP-2 after surgery in spinal cord and DRGs was evaluated using Colorimetric quantitative detection. The results revealed an increase in MMP-9 activity in the DRGs at 24 h and 48 h after subcutaneous remifentanil infusion during surgery in group R as compared with group I (*P* < 0.0001). While the other gelatinase MMP-2, a close family member of MMP-9, did not change significantly after surgery, indicating a unique role of MMP-9 in RIH (Figure 1C, 1D). Notably, no significant change in the activity of MMP-9 or MMP-2 in the lumber spinal cord was observed after intraoperative remifentanil infusion (Figure 1E, 1F). Results of western blotting suggested that the expression of MMP-9 also up-regulated in DRGs in group R (*P* < 0.0001) (Figure 1G–1I).

### Intraoperative remifentanil infusion induced MMP-9 in MOR-expressing DRG neurons

**Figure 2:**
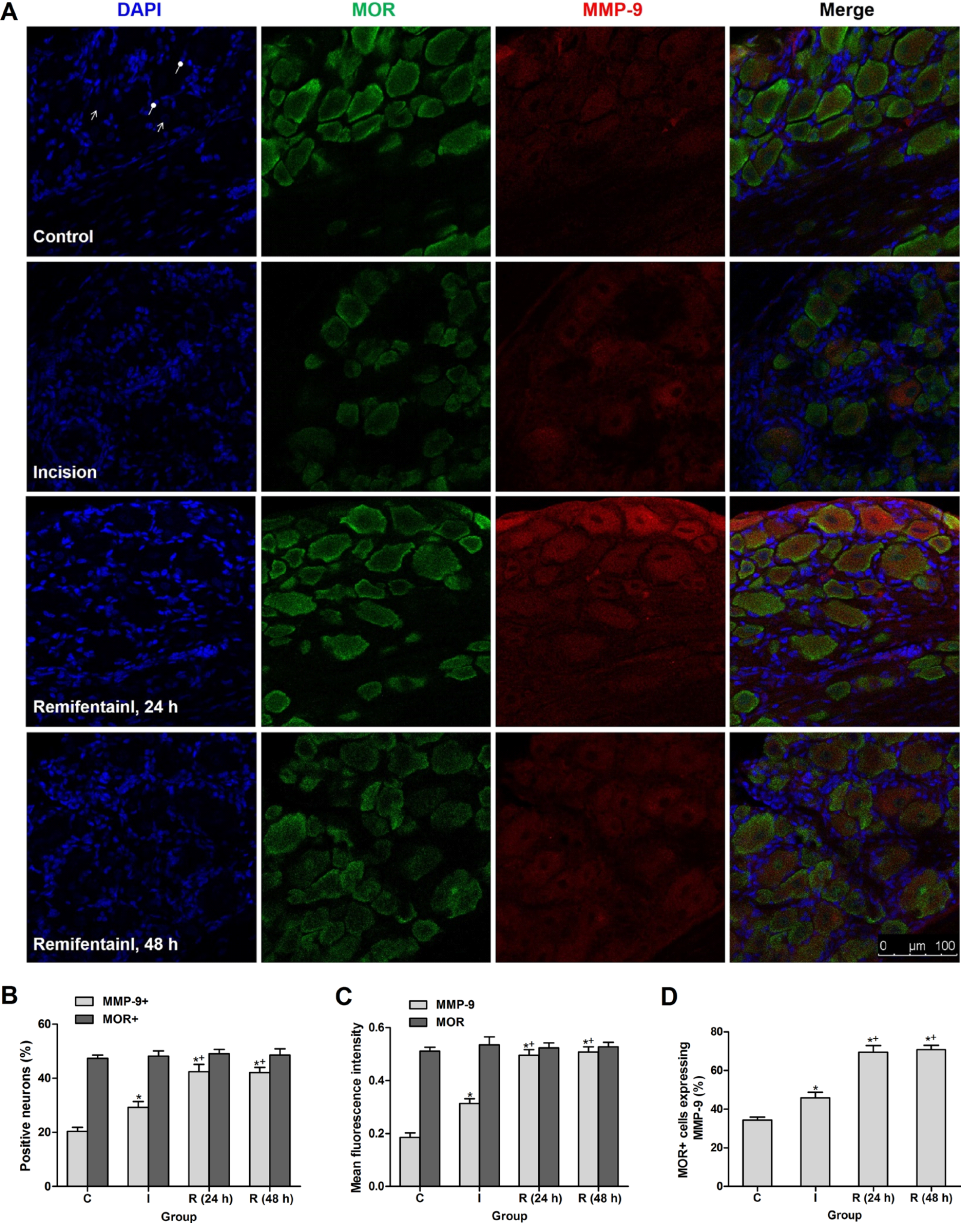
Intraoperative subcutaneous infusion remifentanil-induced MMP-9 up-regulation was enriched in MOR-expressing DRG neurons (**A**) Triple staining showing co-localization of MMP-9 (red), MOR (green) and DAPI (blue) in DRG neurons of control, incisional and remifentanil infused incisional rats. Arrows with triangle head and round head indicated nuclei of neurons (no staining) and SGCs, respectively. (**B**) Percentage of MMP-9 + and MOR + neurons in DRGs at 24 h or 48 h after surgery (*n* = 5). (**C**) Quantification of mean fluorescence intensity of MMP-9 and MOR in DRGs after surgery (*n* = 5). (**D**) Percentage of MOR-positive neurons expressing MMP-9 in DRGs after surgery (*n* = 5). Values expressed as mean ± SD. The treatment style was the same as described in Figure 1. Significant difference was revealed after One-way ANOVA (*P < 0.05 compared with group C, ^+^*P* < 0.05 compared with group I, Bonferroni post hoc tests).

Double immunofluorescence staining showed that MMP-9 was expressed in 20.36% and 29.20% DRG neurons in control rats and incisional rats at 24 h after surgery respectively, and the percentage was significantly increased in group R at 24 h and 48 h after subcutaneous remifentanil infusion during surgery (*P* < 0.0001) (Figure 2A, 2B). The fluorescence intensity of MMP-9 was up-regulated in DRGs in group R (*P* < 0.0001), in support of the Western blotting results. However, the expression of MOR per se did not change in group I or group R after surgery (Figure 2B, 2C). Further analysis demonstrated that the percentage of MOR-positive DRG neurons expressing MMP-9 increased from 45.92% in group I to 69.44% in group R at 24 h after surgery (*P* < 0.0001) (Figure 2D).

### Single intraperitoneal injection of NAC alleviated remifentanil-induced hyperalgesia and suppressed remifentanil-induced activation of MMP-9 in DRGs

**Figure 3:**
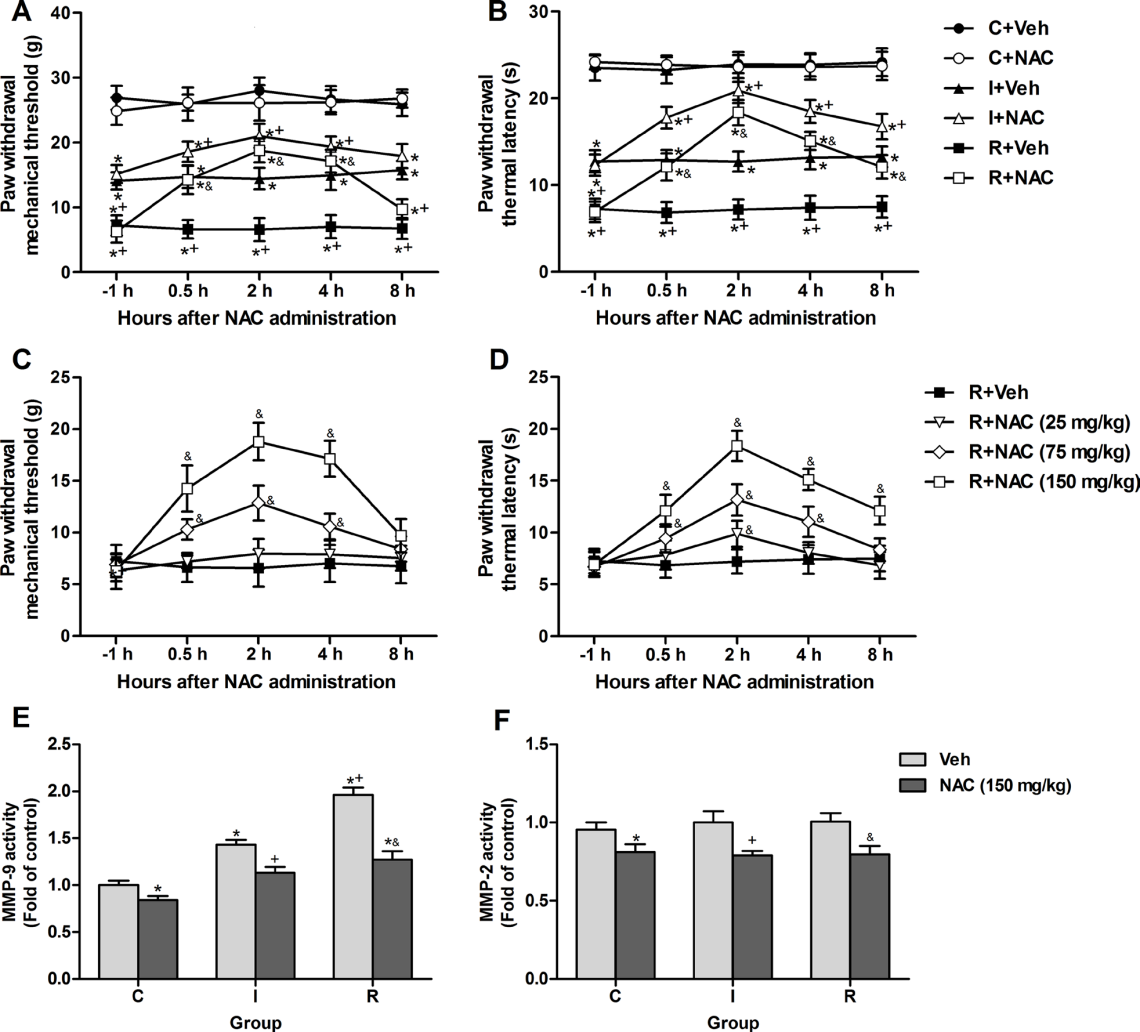
Single intraperitoneal injection of NAC at 24 h after surgery attenuated intraoperative remifentanil-induced hyperalgesia and suppressed the remifentanil-induced activation of MMP-9 (**A** and **B**) NAC (150 mg/kg, i.p.) significantly attenuated mechanical allodynia and thermal hyperalgesia after incision with or without remifentanil infusion (*n* = 8). (**C** and **D**) Effects of various doses of NAC (25, 75, 150 mg/kg, i.p.) on remifentanil-induced mechanical allodynia and thermal hyperalgesia (*n* = 8). (**E** and **F**) The activity of MMP-9 and MMP-2 was inhibited in ipsilateral lumbar DRGs of all groups at 2 h after NAC injection (*n* = 5). Values expressed as mean ± SD. C+Veh: Vehicle (0.9% saline, 1 ml) was injected intraperitoneally in control rats; C+NAC: NAC was injected intraperitoneally in control rats; I+Veh: Vehicle was injected intraperitoneally in incisional rats; I+NAC: NAC was injected intraperitoneally in incisional rats; R+Veh: Vehicle was injected intraperitoneally in incisional rats infused with intraoperative remifentanil; R+NAC: NAC was injected intraperitoneally in incisional rats infused with intraoperative remifentanil. Significant difference in pain behaviors was revealed after Repeated measures ANOVA, and significant difference in the results of Colorimetric quantitative detection was revealed after One-way ANOVA (*P < 0.05 compared with group C+Veh, +*P* < 0.05 compared with group I+Veh, ^&^*P* < 0.05 compared with group R+Veh, Bonferroni post hoc tests).

At 24 h after incisional surgery, the PWMT and PWTL were decreased by 14.09 ± 1.32 g and 12.74 ± 1.29 s in group I, by 7.24 ± 1.56 g and 7.25 ± 1.18 s in group R. Single doses of NAC (25 mg/kg, 75 mg/ kg, 150 mg/ kg) were administrated intraperitoneally to rats after the mechanical threshold and thermal latency tests. NAC (150 mg/kg, i.p.) significantly attenuated pain behaviors in group I (PWMT: *P* = 0.002 at 0.5 h, *P* < 0.0001 at 2 h, *P* = 0.00047 at 4 h, *P* = 0.159 at 8 h; PWTL: *P* < 0.0001 at 0.5 h, 2 h and 4 h, *P* = 0.00023 at 8 h) and group R (PWMT: *P* < 0.0001 at 0.5 h, 2 h and 4 h, *P* = 0.011 at 8 h; PWTL: *P* < 0.0001 at 0.5 h, 2 h, 4 h and 8 h), but exerted no influence on PWMT or PWTL of control rats (Figure 3A, 3B). Moreover, NAC dose-dependently reduced the mechanical allodynia and thermal hyperalgesia induced by intraoperative remifentanil infusion. Compared with group R+Veh, PWMT and PWTL in group R+NAC (150 mg/kg) began to increase at 0.5 h after administration and this increase peaked at 2 h, decreased within 8 h (Figure 3C, 3D). Moreover, NAC (150 mg/kg, i.p.) significantly inhibited the activities of MMP-9 (*P* = 0.042 in control rats, *P* = 0.00013 in incisional rats, *P* < 0.0001 in remifentanil-treated incisional rats) and MMP-2 (*P* = 0.00055 in control rats, *P* = 0.00363 in incisional rats, *P* = 0.00014 in remifentanil-treated incisional rats) at 2 h after injection in the DRGs of rats from all groups (Figure 3E, 3F).

### NAC inhibited remifentanil-induced IL-1β up-regulation via MMP-9 in DRGs

**Figure 4:**
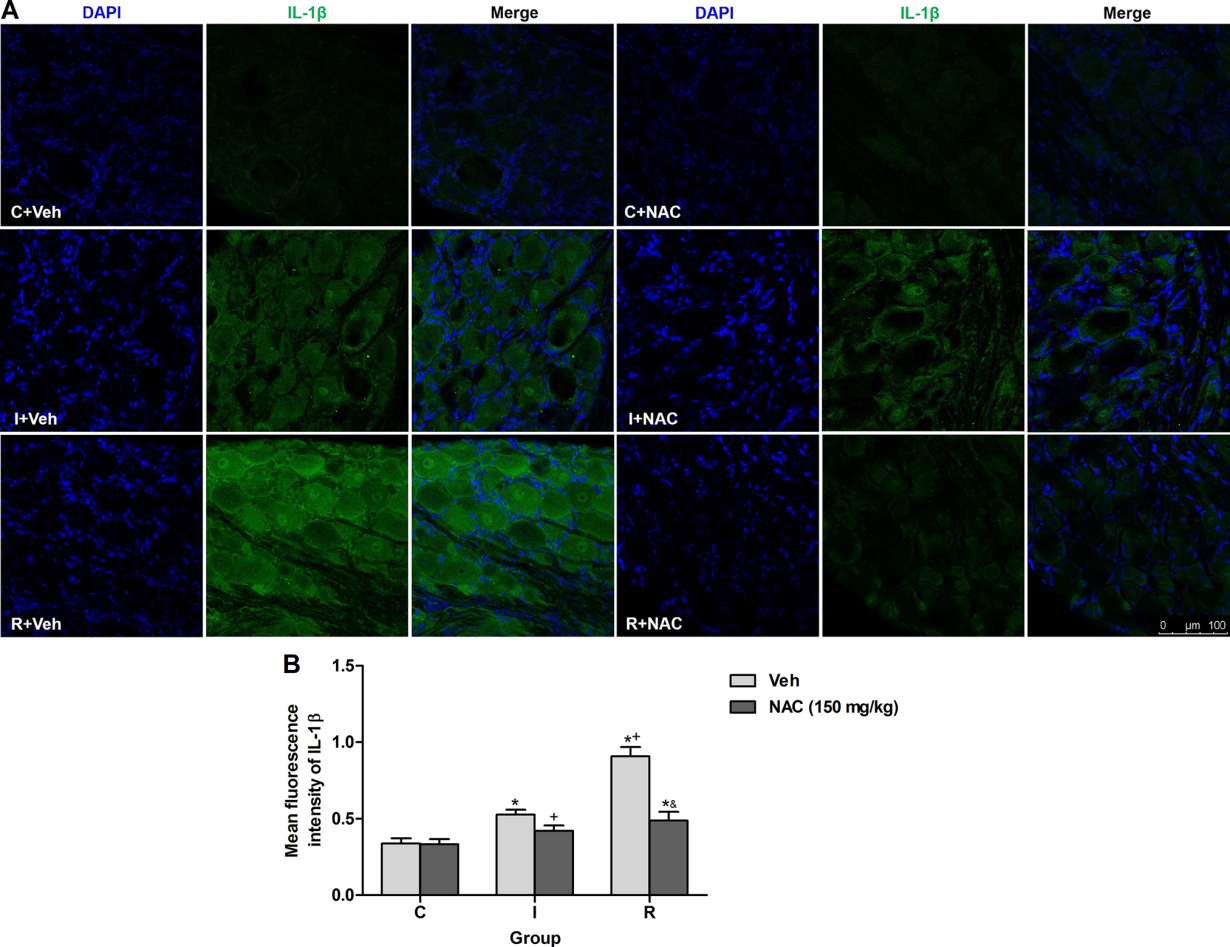
NAC inhibited remifentanil-induced IL-1β up-regulation via MMP-9 in DRGs (**A**) DAPI nuclei staining (left panels, blue), IL-1β immunostaining (middle panels, green) and merge (right panels) in DRGs sections of vehicle or NAC treated rats at 2 h after intraperitoneal injection. (**B**) Quantification of mean fluorescence intensity of IL-1β (*n* = 5). Values expressed as mean ± SD. The treatment style was the same as described in Figure 3. Significant difference was revealed after One-way ANOVA (*P < 0.05 compared with group C+Veh, ^+^*P* < 0.05 compared with group I+Veh, ^&^*P* < 0.05 compared with group R+Veh, Bonferroni post hoc tests).

IL-1β, a critical substrate of MMP-9, is essential for the generation and maintenance of hyperalgesia. Previous study suggested that MMP-9 is required for the acute morphine-induced IL-1β activation in DRGs [19]. Immunofluorescence staining was applied to test whether remifentanil infusion would induce mature active IL-1β (17 kDa) expression via MMP-9 and the effects of NAC in DRGs. Low level of IL-1β expression was observed in DRGs of control rats. The fluorescence intensity of IL-1β was increased significantly in incisional rats with intraoperative remifentanil infusion (*P* < 0.0001). Notably, this increase was alleviated significantly at 2 h after intraperitoneal injection of NAC in incisional rats with or without remifentanil infusion (*P* = 0.011 in incisional rats, *P* < 0.0001 in remifentanil-treated incisional rats). However, NAC did not alter the expression of IL-1β in control rats. Staining for DAPI (nuclear marker) in DRGs indicated the localization of SGCs, while no staining indicated neurons in DRGs. The cellular localization of IL-1β was found in cytoplasm of both SGCs and neurons of remifentanil-infused rats (Figure 4A, 4B).

### NAC inhibited remifentanil-induced spinal MAPK family phosphorylation and glial activation in ipsilateral spinal cord dorsal horn

**Figure 5:**
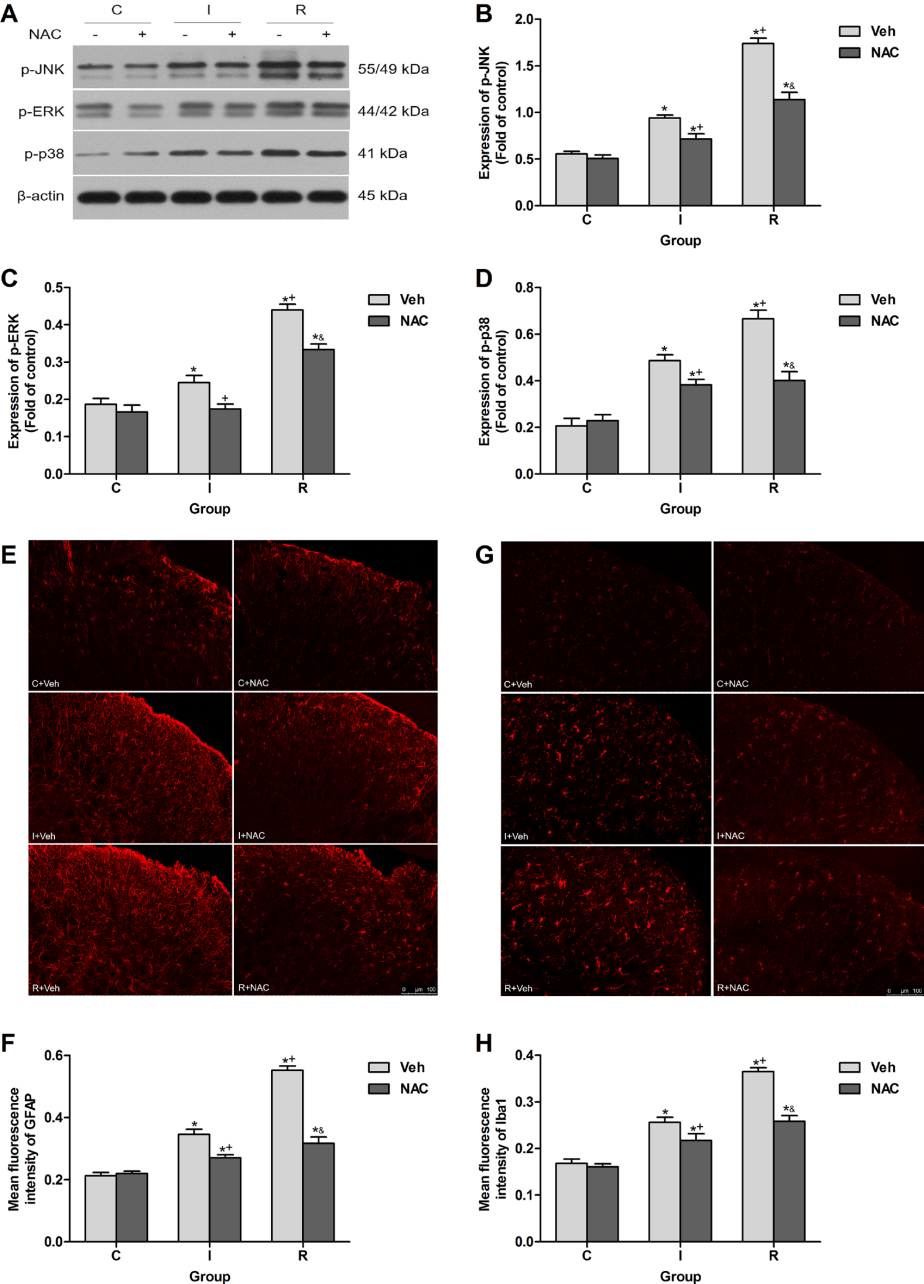
NAC inhibited remifentanil-induced spinal MAPK family phosphorylation and glial activation in ipsilateral spinal cord dorsal horn (**A**) Western blotting for p-JNK, p-ERK, p-p38 and β-actin resulted in products of 55/49, 44/42, 41 and 43 kDa. (**B–D**) Densitometric quantification of p-JNK, p-ERK and p-p38 immunoreactivity on Western blots. β-actin was used as a loading control (*n* = 5). (**E** and **F**) Images and quantification of immunofluorescence showing GFAP in the ipsilateral dorsal horns. (**G** and **H**) Images and quantification of immunofluorescence showing Iba1 in the ipsilateral dorsal horns. Quantification of immunofluorescence was presented as mean fluorescence intensity in the superficial dorsal horns (*n* = 5). The ipsilateral lumbar spinal cord was collected and analyzed 2 h after intraperitoneal injection. Values expressed as mean ± SD. The treatment style was the same as described in Figure 3. Significant difference was revealed after One-way ANOVA (*P < 0.05 compared with group C+Veh, ^+^*P* < 0.05 compared with group I+Veh, ^&^*P* < 0.05 compared with group R+Veh, Bonferroni post hoc tests).

IL-1β produced and cleaved in DRGs may elicit hypersensitivity in spinal dorsal horn via causing hyperexcitability of primary sensory neurons by increasing sodium currents and suppressing potassium currents [24–26]. The activation (phosphorylation) of MAPK family in spinal cord contributes to the central hypersensitivity, here effects of NAC on the phosphorylation levels of MAPKs in ipsilateral lumber spinal cord dorsal horn at 2 h after injection were detected. NAC (150 mg/kg) reduced the phosphorylation of c-Jun N-terminal kinase (JNK), extracellular regulating kinase (ERK) and p38 induced by intraoperative remifentanil infusion (*P* < 0.0001) (Figure 5A–5D).

Spinal glial activation, which may be triggered by MMP-9 produced in DRGs [17], is essential dynamic regulators of neuronal network and inflammation in RIH [7]. Results of immunofluorescence indicated that single injection of NAC inhibited the up-regulation of glial fibrillary acidic protein (GFAP, marker for astrocyte) and ionized calcium-binding adapter molecule 1 (Iba1, marker for microglia) in ipsilateral spinal cord dorsal horn after intraoperative remifentanil infusion (*P* < 0.0001) (Figure 5E–5H).

### NAC inhibited remifentanil-induced PKCγ phosphorylation and NR1, NR2B phosphorylation in ipsilateral spinal cord dorsal horn

**Figure 6:**
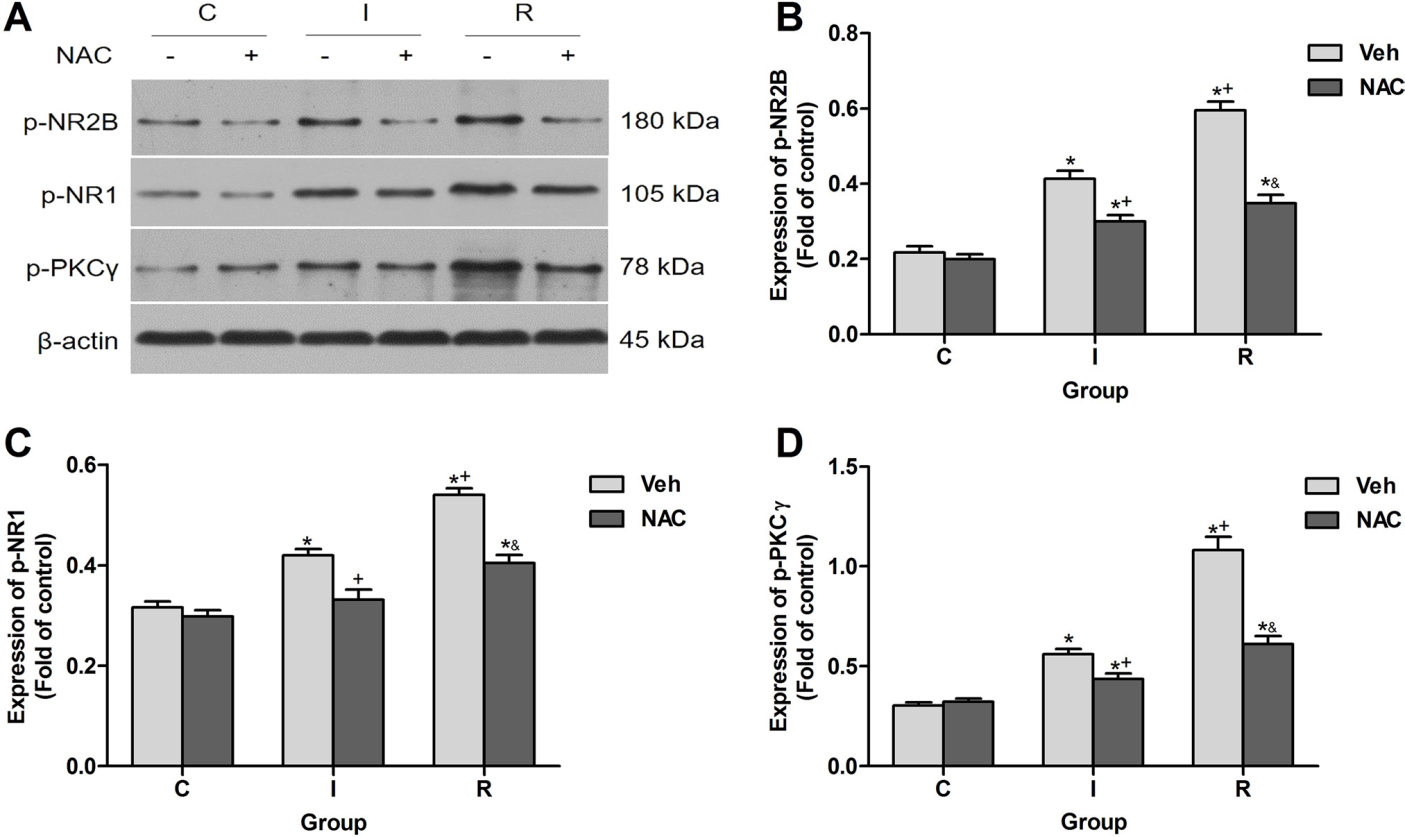
NAC inhibited remifentanil-induced NR2B phosphorylation, NR1 phosphorylation and PKCγ phosphorylation in ipsilateral spinal cord dorsal horn (**A**) Western blotting for p-NR2B, p-NR1, p-PKCγ and β-actin resulted in products of 180, 105, 78 and 43 kDa. (**B–D**) Densitometric quantification of p-NR2B, p-NR1, p-PKCγ immunoreactivity on Western blots. β-actin was used as a loading control (*n* = 5). The ipsilateral lumbar spinal cord was collected and analyzed 2 h after intraperitoneal injection. Values expressed as mean ± SD. The treatment style was the same as described in Figure 3. Significant difference was revealed after One-way ANOVA (*P < 0.05 compared with group C+Veh, ^+^*P* < 0.05 compared with group I+Veh, ^&^*P* < 0.05 compared with group R+Veh, Bonferroni post hoc tests).

Sensitization of nociceptive pathways has been considered mainly a neurocentric plastic mechanism in spinal dorsal horn. The phosphorylation and activation of NMDAR in spinal neurons enhanced sensory responses in RIH [4]. Effects of NAC on the neuronal activation induced by remifentanil in ipsilateral lumber spinal cord dorsal horn at 2 h after injection were also detected. Results of western blotting showed that NAC dramatically decreased the remifentanil-induced phosphorylation of PKCγ, NR1 and NR2B (*P* < 0.0001) (Figure 6).

## DISCUSSION

The major findings of the present study were as follows: (1) intraoperative remifentanil infusion induced MMP-9 up-regulation and activation in primary sensory neurons expressing MOR in DRGs, but not in spinal cord; (2) single intraperitoneal injection of NAC attenuated remifentanil-induced postoperative mechanical allodynia and thermal hyperalgesia via suppressing remifentanil-induced activation of MMP-9 in DRGs of incisional rats; (3) NAC also inhibited remifentanil-induced cleavage of IL-1β in DRGs; (4) NAC significantly inhibited remifentanil-induced glial activation and phosphorylation of MAPK family, PKCγ, NR1 and NR2B in ipsilateral spinal cord dorsal horn. Based on these findings, we postulate that up-regulation and activation of neuronal MMP-9, and subsequent IL-1β cleavage in DRGs following intraoperative remifentanil infusion triggers glial activation and sensory neuron excitability in spinal cord dorsal horn, promoting the development of RIH.

The gelatinase MMP-9 appears to result in opioid-elicited paradoxical pro-nociceptive effects. Recent studies indicated that up-regulation and activation of MMP-9 in spinal cord was implicated in the physical dependence on chronic morphine through possible neuronal activation and interaction with NR1 and NR2B [27]. Repeated morphine treatment-induced tolerance was associated with a persistent MMP-9 activation after the transient increment in MMP-9 expression in midbrain [28]. Acute subcutaneous morphine induced a significant up-regulation of MMP-9 in MOR-positive neurons in DRGs [20], sabotaging morphine-induced analgesia via MMP-9-triggered peripheral neuronal-glial interactions, specifically, MMP-9-dependent activation of satellite glial cells and cleavage of IL-1β [19]. Moreover, MMP-9 also mediated neuropathic pain via degenerative and pro-inflammatory mechanisms. Nerve injury induced an early rapid increase of active MMP-9 in DRGs by 24 h [29], and in spinal cord by 14 d [18]. Active MMP-2 in DRGs and in spinal cord was delayed until 7 d and persisted through 21 d [18]. The different time pattern suggested distinct roles of MMP-9 and MMP-2 in the development and maintenance of pain. Our results indicated that an increase in MMP-9 activity and expression in DRGs following intraoperative remifentanil infusion was correlated with remifentanil-evoked pro-nociceptive actions. However, no increase in MMP-2 activity after remifentanil infusion was observed, in support of previous studies that MMP-2 was not involved in early-phase development of hyperalgesia.

Recent evidence suggested that subcutaneous morphine increased MMP-9 expression and activity in DRGs, and this effect was inhibited by opioid receptor antagonist naloxone and the selective MOR antagonist CTAP [20]. Activation of MAPKs pathways following morphine treatment may contribute to the MOR-dependent MMP-9 expression in DRG neurons [30]. Our immunofluorescence results showed that intraoperative remifentanil infusion-induced MMP-9 increase was enriched in MOR-positive DRG neurons, also supported a MOR-dependent mechanism of MMP-9 induction in DRGs.

These results indicated that MMP-9 may be a vital target in the therapeutic intervention of RIH which occurs mainly in the early-phase after intraoperative remifentanil infusion. Previous reports suggested that gene knockout of MMP-9 or administration of inhibitors for general or specific inhibition of MMP-9 and MMP-2 reduced nociceptive pain behaviors induced by peripheral nerve injury [17, 31]. However, considering that the normal physiological effects of MMPs are very essential [32], MMP-9 gene knockout may exhibit various adverse effects, and inhibitors of MMPs in animal researches have not been applied to clinical use. A recent study used NAC, a compound with abundant cysteine residues, with negligible adverse effects and low cost, to target the ‘cysteine switch’ of MMPs, and demonstrated that NAC significantly reduced the activity of MMP-9 and MMP-2 with inhibition efficiencies of 86.3% and 70.8% *in vitro*, 62.0% and 47.3% *in vivo* respectively, subsequently attenuated chronic constrictive injury-induced mechanical allodynia and thermal hyperalgesia [18]. Therefore, we used NAC, an effective drug with excellent clinical safety history, which was injected intraperitoneally, to inhibit the activity of MMP-9 in DRGs. In accordance with the Colorimetric quantitative detection results that NAC significantly reduced intraoperative remifentanil-induced activation of MMP-9 in DRGs, behavioral tests revealed that NAC effectively attenuated remifentanil-induced postoperative hyperalgesia.

MMP-9 has been proven to cleave IL-1β, which is one of the most important pro-inflammatory cytokines in nervous system and promotes the development of opioid-induced tolerance and hyperalgesia [33]. Our data demonstrated that intraoperative remifentanil infusion induced the up-regulation of mature active IL-1β in both DRG neurons and SGCs, and NAC decreased remifentanil-induced IL-1β activation via inhibiting MMP-9. It was suggested that the cleavage of IL-1β required MMP-9 which was released from DRG neurons expressing MOR following remifentanil infusion, retained in extracellular space between neurons and SGCs, and mediated neuronal-glial interactions. Similarly, previous studies showed that intrathecal injection with MMP-9 caused IL-1β cleavage in DRGs accompanied by significant allodynia [17]. Primary sensory neurons particularly nociceptors in DRGs express IL-1 receptors (IL-1R), and activation of IL-1R by IL-1β can activate nociceptors rapidly to generate action potentials and elicit pain hypersensitivity via regulating sodium and potassium currents [25, 26]. Since the acute opioid-induced increase in IL-1β occurred primarily in DRGs, down-regulation of IL-1β in DRGs using selective siRNA potentiated and prolonged morphine-induced analgesia [19].

Furthermore, MMP-9 and MMP-2 produced in DRG neurons may trigger the activation of microglia and astrocytes in spinal dorsal horn co-localized with phosphorylated MAPKs and contribute to neuropathic pain through increasing IL-1β cleavage [17]. Inhibiting IL-1β signaling with a neutralizing antibody or antagonist prevented the hyperalgesia [17, 34, 35], establishing that IL-1β as a downstream regulator of MMP-9 mediated the nociceptive information from DRGs to spinal cord. The phosphorylation of MAPK family and the activation of glial cells in spinal dorsal horn induced by intraoperative remifentanil infusion can result in the production and release of multiple inflammatory mediators, and produce RIH [7, 36–38]. In addition to glia, the phosphorylation and activation of NMDAR in spinal neurons also resulted in the synaptic plasticity and enhanced sensory responses after intraoperative remifentanil infusion [4, 39–41]. The present study found that NAC can effectively decrease the phosphorylation of NR1, NR2B and MAPK family, decrease sensory neuron excitability and inhibit glial activation in spinal dorsal horn caused by remifentanil infusion.

In addition to the direct inhibition of the activation of MMP-9 in DRGs as revealed by Colorimetric quantitative detection in this study, previous studies indicated that NAC may alleviate neuropathic pain and inflammatory pain through reducing nitric oxide metabolites [42], scavenging reactive oxygen species (ROS) [43], and enhancing endogenous activation of type-2 metabotropic glutamate receptors (mGluR) in spinal cord [44]. The gelatin lytic activity and expression of MMP-9 could also be abolished indirectly by NAC via its ROS scavenging effect [45]. Moreover, inhibition of mGluR2/3 or system Xc^-^ could only mildly suppress NAC's inhibition of MMP-9 activity and partly reduce NAC's inhibition of mechanical allodynia in a rat model of neuropathic pain [18]. Therefore, MMP-9 may be a more vital target of NAC in the treatment of pain.

In conclusion, intraoperative remifentanil infusion elicited MMP-9 activation and up-regulation in primary sensory neurons and subsequently caused IL-1β cleavage in DRGs, leading to the central sensitization in spinal cord to induce postoperative hyperalgesia. The safe drug NAC, which was effective in inhibiting MMP-9 activity, significantly attenuated RIH. These findings may represent a bright prospect for the treatment of RIH.

## MATERIALS AND METHODS

### Animals

Adult male Sprague-Dawley rats, weighing 220–250 g, were provided by the Laboratory Animal Center of Drum Tower Hospital. Rats were housed in climate-controlled rooms (22 ± 2°C) on a 12-hour light/dark cycle and with free access to food and water. Rats were acclimatized for 1 week before experimental procedures. All experimental procedures were approved by the Institutional Animal Care and Use Committee in Nanjing University and were in accordance with the ethical guidelines for the use of laboratory animals. Efforts were made to minimize rats suffering. The sample size was calculated using a statistical power analysis, and was selected according to previous reports, to use the minimum number of rats necessary to obtain valid results.

### Drug preparation

Remifentanil hydrochloride (RenFu Co., Yichang, China) and NAC (Sigma, St. Louis, MO) were dissolved in 0.9% saline solution, respectively. Remifentanil (0.04 mg/ kg, 0.4 ml) was infused subcutaneously during surgical incision over a period of 30 min using an apparatus pump. The infusion rate was 0.8 ml/h. Rats in group C and group I received the same volume of saline in identical conditions. Single intraperitoneal (i.p.) administration of NAC (25 mg/kg, 75 mg/kg, 150 mg/kg) in a volume of 1 ml was performed at 24 h after plantar incision. The dosages of NAC were chosen referring to the previous study [18] and our preliminary experiments. For vehicle treatment, a same volume of 0.9% saline was intraperitoneally injected.

### Incisional surgery

Rats in group I and group R were anesthetized with sevoflurane (induction, 3%, surgery, 1%; Heng Rui Co., Shanghai, China) via a nose mask. The right hindpaw was placed through a hole in a sterile drape, and the plantar aspect was sterilized with 5% povidone-iodine solution. A longitudinal 1-cm incision was made through the skin and fascia, starting at 0.5 cm from the edge of the heel and extending toward the toes of the right hindpaw. The plantar muscle was elevated using forceps and incised longitudinally, leaving the muscle origin and insertion intact. After hemostasis with gentle pressure, the skin was closed with two mattress sutures of 5–0 nylon. The wound site was covered with Aureomycin ointment. Control rats underwent a sham procedure that consisted of administration of sevoflurane and subcutaneous infusion of the same volume of saline without incision [46].

### Pain behavioral assessment

Pain behavioral tests were performed at 24 h before surgery (baseline), 2 h, 6 h, 24 h, 48 h after surgery, and before NAC administration (24 h after surgery), 0.5 h, 2 h, 4 h, 8 h after NAC administration (*n* = 8 rats per group). All tests were performed during the light phase. Before each test, rats were allowed to acclimatize for at least 30 min. All experiments were performed by the same investigators in a quiet test room.

### Paw withdrawal mechanical threshold (PWMT)

Mechanical allodynia was assessed using a Dynamic Plantar Analgesiometer (Ugo Basile, Varese, Italy). Each rat was placed into individual transparent plexiglass compartments (20 cm × 25 cm × 15 cm) onto a metal mesh floor (graticule: 1 cm × 1 cm). A metal wire was pressed vertically against the central plantar surface and the force was increased uniformly. A positive response was defined as paw flinching or withdrawal of the hindpaw. The test was repeated five times with a 5-min interval between each application of force.

### Paw withdrawal thermal latency (PWTL)

Thermal hyperalgesia to radiant heat was determined using an automatic Plantar Test (Hargreaves Apparatus, Ugo Basile, Varese, Italy) according to a previous method [47]. Rats were placed into individual transparent plexiglass compartments on a clear elevated glass floor. The movable heat stimulator was moved to focus onto the central plantar surface of the hindpaw through the glass plate. The nociceptive end points in the radiant heat test were the characteristic lifting or licking of the hindpaw, and the time to the end point was considered PWTL. A cutoff time of 25 seconds as used to avoid tissue damage. There were 5 trials per rat and 5-minute intervals between trials.

### Colorimetric quantitative detection of MMP-2/9 activity

Rats were anesthetized deeply with 5% sevoflurane, the L4-L5 right DRGs and right spinal cord dorsal horn segments were removed rapidly and stored in liquid nitrogen (*n* = 5 rats per group). The activity of MMP-2 and MMP-9 was determined using Colorimetric quantitative detection kits (Genmed Scientifics, MA, USA) according to the manufacture's protocol. Briefly, protein samples were ground in liquid nitrogen, incubated in lysis buffer on ice, centrifuged at 10000 g for 10 min at 4°C and the supernatant was obtained. The protein concentration was determined using the BCA method. 100 μg of protein was added to the reaction buffer in a cuvette. Then, the substrate was added. The absorption at 412 nm was measured using a spectrophotometer during a period of 15 min. The fold-increase in MMP-2 and MMP-9 activity was determined by comparing the results of treated samples with the level of untreated control.

### Western blotting

The right DRGs and right dorsal horn of spinal cord segments were removed rapidly and stored in liquid nitrogen under deep anesthesia (*n* = 5 rats per group). Tissue samples were homogenized in ice-cold lysis buffer. The homogenate was centrifuged at 13000 rpm for 10 min at 4°C and supernatant was obtained. Protein lysates (50 μg) and protein molecular weight marker were separated using SDS-PAGE (10%) for 90 min at 120 V, and transferred onto polyvinylidene difluoride membranes (Millipore Corporation, MA, USA) at 200 mA for 2 h. After blocked in skim milk for 2 h at room temperature, the membranes were incubated with the primary antibodies overnight at 4°C, including mouse anti-MMP-9 (1:1000, Abcam, Cambridge, UK), mouse anti-MMP-2 (1:3000, Abcam, Cambridge, UK), rabbit anti-p-ERK1 (pT202/pY204)+ERK2 (pT185/pY187) (1:2000, Abcam, Cambridge, UK), rabbit anti-p-JNK1+JNK2 (pT183+pY185, 1:1000, Abcam, Cambridge, UK), rabbit anti-p-p38 (phospho Y182, 1:1000, Abcam, Cambridge, UK), rabbit anti-p-PKCγ (phospho T514, 1:1000, Abcam, Cambridge, UK), rabbit anti-p-NR1 (phospho S896, 1:1000, Abcam, Cambridge, UK), rabbit anti-p-NR2B (phospho Y1472, 1:1000, Abcam, Cambridge, UK), and rabbit anti-β-actin antibody (1:2000, Cell Signaling Technology, Danvers, MA). The membranes were washed with TBST buffer for 1 h and incubated with the secondary antibody conjugated with horseradish peroxidase (1:10000, Abcam, Cambridge, UK) for 2 h at room temperature and visualized in ECL solution followed by film exposure for 1–10 min. β-actin was used as a loading control for total protein. The density of specific bands was measured using an analysis system (Quantity One V4.31, Bio-Rad, Hercules, CA).

### Immunofluorescence

Rats were deeply anaesthetized with sevoflurane and perfused through the ascending aorta with physiological saline, followed by 4% paraformaldehyde in 0.1 M phosphate buffer at pH 7.2-7.4, 4°C. After perfusion, the L4-L5 right DRGs and lumbar spinal cord segments were removed and postfixed in the same fixative for 6 h and then dehydrated in 30% sucrose (*n* = 5 rats per group). Sections (20 μm) were cut in a freezing microtome. After washed in PBS, the DRG and spinal cord sections were blocked with 100% (v/v) goat serum or 50% (v/v) donkey serum with 0.3% Triton X-100 respectively for 2 h at room temperature. Some DRG sections were incubated over two nights at 4°C with a mixture of primary antibodies in 10% goat serum: rabbit anti-MOR (1:200, Abcam, Cambridge, UK) and mouse anti-MMP-9 (1:100, Abcam, Cambridge, UK). Other DRG sections were incubated over two nights at 4°C with primary antibodies in 10% goat serum: rabbit anti-IL-1β (1:100, Abcam, Cambridge, UK). The spinal cord sections were incubated over two nights at 4°C with the following primary antibodies: mouse anti-GFAP (1:300, Cell Signaling Technology, Danvers, MA) in 10% goat serum and goat anti-Iba1 (1:500, Abcam, Cambridge, UK) in 10% donkey serum, respectively. After washed in PBS, the DRG sections were incubated for 2 h at room temperature with a mixture of secondary antibodies in 10% goat serum: FITC-conjugated goat anti-rabbit antibodies (Alexa Fluor 488, 1:500, Invitrogen, Carlsbad, CA, USA) and goat anti-mouse secondary antibodies (Alexa Fluor 594, 1:500, Invitrogen, Carlsbad, CA). The spinal cord sections were incubated for 2 h at room temperature with secondary antibodies respectively: goat anti-mouse secondary antibodies (Alexa Fluor 594, 1:500, Invitrogen, Carlsbad, CA) and donkey anti-goat secondary antibodies (Alexa Fluor 555, 1:1500, ThermoFisher, Waltham, MA). DAPI (4′,6-diamidino-2-phenylindole; Sigma, St. Louis, MO) staining was used to determine the cell nuclei. The stained sections were washed in PBS, and mounted on glass slides, air-dried and coverslipped with Fluoromount G (Fisher Scientific, Ottawa, Canada). Images at ×200 magnification were captured using a confocal microscope (Leica TCS SP2; Leica, Wetzlar, Germany).

### Statistical analysis

Data were expressed as the mean ± SD. SPSS 15.0 (SPSS Inc., Chicago, IL) was used to conduct all the statistical analyses. Rats were assigned to different treatment groups in a randomized manner. Multiple comparisons were carried out to determine the overall differences of pain behaviors at each time point and repeated measures analysis of variance (ANOVA) was performed to assess the changes of pain behaviors over time. One-way ANOVA was used to determine differences in the results of Colorimetric quantitative detection of MMP-2/9 activity, western blotting and immunofluorescence among groups. In both cases, when significant main effects were observed, Bonferroni post hoc tests were conducted to determine the source(s) of these differences. *P* < 0.05 was considered statistically significant.

## Abbreviations

α7-nAchR: alpha-7 nicotinic acetylcholine receptor
ANOVA: analysis of variance
Cdk5: cyclin-dependent kinase 5
CXCR2: chemokine (C-X-C motif) receptor 2
DRG: dorsal root ganglia
ECM: extracellular matrix
ERK: extracellular regulating kinase
GSK-3β: glycogen synthase kinase-3β
IL: interleukin
i.p.: intraperitoneal
JNK: c-Jun N-terminal kinase
MAPK: mitogen-activated protein kinase
mGluR: metabotropic glutamate receptors
MMP: Matrix metalloproteinase
MOR: mu-opioid receptor
NAC: N-acetyl-cysteine
NMDAR: N-methyl-d-aspartate receptor
NR1: NMDA receptor subunit 1
NR2B: NMDA receptor subunit 2B
PKCγ: protein kinase Cγ
PWMT: Paw withdrawal mechanical threshold
PWTL: Paw withdrawal thermal latency
RIH: remifentanil-induced hyperalgesia
ROS: reactive oxygen species
SGCs: satellite glial cells
TNF-α: tumor necrosis factor-α
